# The Comfort and Measurement Precision-Based Multi-Objective Optimization Method for Gesture Interaction

**DOI:** 10.3390/bioengineering10101191

**Published:** 2023-10-13

**Authors:** Wenjie Wang, Yongai Hou, Shuangwen Tian, Xiansheng Qin, Chen Zheng, Liting Wang, Hepeng Shang, Yuangeng Wang

**Affiliations:** 1 School of Mechanical Engineering, Inner Mongolia University of Science and Technology, Baotou 014010, China; gua19891128@126.com (L.W.); 2022022277@stu.imust.edu.cn (H.S.); 2022022261@stu.imust.edu.cn (Y.W.); 2 Inner Mongolia Firmaco HongYuan Electric Co., Ltd., Baotou 014010, China; 3 Inner Mongolia North Heavy Industries Group Co., Ltd., Baotou 014010, China; 13224872669@163.com; 4 School of Mechanical and Electrical Engineering, Northwestern Polytechnical University, Xi’an 710072, China; xsqin@nwpu.edu.cn (X.Q.); chen.zheng@nwpu.edu.cn (C.Z.)

**Keywords:** gesture interaction, multi-objective optimization, comfort, measurement precision

## Abstract

As an advanced interaction mode, gestures have been widely used for human–computer interaction (HCI). This paper proposes a multi-objective optimization method based on the objective function $J_{CP}$ to solve the inconsistency between the gesture comfort $J_{CS}$ and measurement precision $J_{P_{H}}$ in the gesture interaction. The proposed comfort model CS takes seventeen muscles and six degrees of freedom into consideration based on the data from muscles and joints, and is capable of simulating the energy expenditure of the gesture motion. The CS can provide an intuitive indicator to predict which act has the higher risk of fatigue or injury for joints and muscles. The measurement precision model ${∆P}_{H}$ is calculated from the measurement error $\left({∆X}_{H},{∆Y}_{H},{∆Z}_{H}\right)$ caused by calibration, that provides a means to evaluate the efficiency of the gesture interaction. The modeling and simulation are implemented to analyze the effectiveness of the multi-objective optimization method proposed in this paper. According to the result of the comparison between the objective function $J_{CS}$, based on the comfort model CS, and the objective function $J_{P_{H}}$, based on the measurement precision models ${∆P}_{H}$, the consistency and the difference can be found due to the variation of the radius $r_{B\_RHO}$ and the center coordinates $P_{B\_RHO}\left(x_{B\_RHO},y_{B\_RHO},z_{B\_RHO}\right)$. The proposed objective function $J_{CP}$ compromises the inconsistency between the objective function $J_{CS}$ and $J_{P_{H}}$. Therefore, the multi-objective optimization method proposed in this paper is applied to the gesture design to improve the ergonomics and operation efficiency of the gesture, and the effectiveness is verified through usability testing.

## 1. Introduction

Human–computer interaction (HCI) examines how people interact with computer systems. It makes use of an interactive way to realize the information flow between computers and people. Traditional HCI is machine-centric, requiring users to become accustomed to the workings of the computer through the use of command languages, graphical user interfaces, and physical interaction equipment. It is urgently necessary to create natural, comfortable, and effective natural interaction technologies in order to circumvent and eliminate the limitations of these regulations. Instead of mechanically converting their operating intentions into specific instructions that the machine can understand, operators can express their willingness to interact naturally as if they were speaking to people thanks to natural interaction technology [1]. This is in contrast to the traditional method of interaction. Gesture interaction, voice interaction, brain–computer interaction, emotional interaction, etc. are some examples of the natural interactions that have emerged in the area of human–computer interaction in recent years [2,3]. In addition to having powerful ideographic capabilities, gestures—a kind of interactive communication that is frequently utilized in social interactions—also exhibit the qualities of intuition, simplicity, and vividness. The state-of-the-art techniques for large gestures and speech recognition in human–computer interaction systems, such as STF and 2DCNN + BiLSTM [4], SAM-SLR [5], Ensemble-NTIS [6], MViT-SLR [7], and FE + LSTM [8], are evaluated using the widely used well-known LRW [9] and AUTSL [10] datasets. Ryumin et al. [4] proposed a benchmark methodology on two well-known datasets: LRW for audio-visual speech recognition and AUTSL for gesture recognition. The accuracy of gesture recognition is achieved through the use of a unique set of spatio-temporal features, including those that take into account lip articulation information. Jiang et al. [5] proposed a novel Skeleton Aware Multimodal SLR framework (SAM-SLR) to take advantage of multi-modal information towards a higher sign language recognition rate. Furthermore, regarding emotion recognition in human–computer interaction, the EEG dataset [11] is trained with differential entropy features extracted from multichannel EEG data; the deep belief networks (DBNs) are introduced to construct EEG-based emotion recognition models for three emotions: positive, neutral, and negative. AffectNet [12] is by far the largest database of facial expressions, enabling further progress in the automatic understanding of facial behavior in both categorical and continuous dimensional space. On AffectNet datasets, Mao et al. [13] proposed POSTER++ that achieves the state-of-the-art FER performance while greatly reducing the parameters and floating point operations of POSTER. She et al. [14] proposed the DMUE method to address the problem of annotation ambiguity from two perspectives: the latent Distribution Mining and the pairwise Uncertainty Estimation. All in all, academics are paying more and more attention to natural interactions technologies.

The study of gesture interaction technology covers a wide range of research topics such as theories and methods of gesture recognition, gesture comfort, gesture design, and usability evaluation [15], as well as application research and development in mobile computing, virtual reality [16], etc. Gestures can be translated into control commands for interactive activities in many fields, such as teleoperation, robotics, virtual reality, education, and entertainment [17], which can benefit from its use. However, long-term human–computer interactions will result in muscle fatigue, low operational efficiency, and operator frustration [18]. Therefore, in order to improve the level of ergonomics and usability of the gesture interaction, it is crucial to study and analyze the gesture comfort, gesture measurement accuracy, and gesture multi-objective optimization for an improved level of ergonomics and usability of the gesture interaction. According to ergonomics research, different gestures have different comfort levels for the operator [18]. In addition, due to the effect of measurement accuracy, different gestures will obtain different gesture recognition rates [19], thus affecting the efficiency and experience of gesture interaction. This research has general applicability to gesture interaction; it would be a valuable study to analyze the relevance of different gestures with assistive technologies.

The gesture comfort is a crucial indicator used to assess the ergonomics of human–computer interaction applications and refers to the level of comfort that employees experience when engaging with their job and the environment. The comfort level is challenging to identify and quantify since it is a subjective experience that varies with the length of time and mood of the human–computer contact process. Comfortable gesture interaction will significantly lessen operator fatigue, increase work time, and enhance the effectiveness and experience of the interactions. In general, there are four criteria used to evaluate human comfort: (1) joint angle range of motion (ROM)-based comfort models, such as RULA [18], LUBA [20], REBA [21], OCRA [22], OCRA-CL [22], and others. These models are based on ROM to evaluate the gesture comfort and only take into account comfort evaluation in a static posture; (2) a comfort model based on ROM and motion data, such as those proposed by Andreoni [23], Ramona [24], etc., which can evaluate the comfort of dynamic gestures but contains less information on the biomechanics of humans; (3) a comfort model based on simulation software is used, for instance, by Keyvani [25], Qing [26], and others to evaluate the human comfort, but standardized software tools have limitations such as not understanding the internal principles of the model and being unable to modify in accordance with the specifications; (4) a comfort model based on sensor-based measurement tools, such as pressure, temperature, and cardiopulmonary function sensors [27,28,29] to measure pressure, temperature, and energy consumption as well as other data indicators to evaluate how comfortable and acceptable a human body is.

The comfort of the human body can be enhanced and improved, and the operator’s workload can be decreased in accordance with the established comfort model, which has practical implications for increasing the ergonomics and HCI efficiency. Sam [30] summarizes the state-of-the-art of research on biomechanical optimization and ergonomic risk assessment utilizing wearable sensors for industrial and sports use, which offers a wealth of information for our study. A multi-objective optimization strategy for gestures based on human intuition, comfort, and gesture recognition rate was proposed by Stern [31,32]. The three indicators of human intuition, comfort, and gesture recognition rate are coordinated by using the optimization calculation approach, although this method is only employed for hand gestures, and experimental analysis and verification have not been carried out. Herman [33] optimizes the gesture comfort during surgical procedures to enhance the ergonomics and operational stability of surgical procedures, but the comfort model utilized does not include biomechanical information. A pilot helmet design strategy that maximizes comfort [34] through pressure distribution and eye position has been reported. Battini [35] et al. predicted the energy expenditure of the human body and assessed the degree of comfort of the human body using gender, height, load, arm position, speed, and duration of action; however, the energy expenditure computed using this method contains less biomechanical information. In order to prevent the overload of nursing staff, Zhang [36] employed the metabolic energy expenditure module to compute the work energy consumption that directly reflects the energy consumption, physical condition, and fatigue recovery time of nursing staff in each sub-task. A human energy expenditure model based on heat dissipation [37] and muscular mechanical energy expenditure [38] was proposed, but it has not been used in a human comfort study. Additionally, there is numerous research on how to make the human body more comfortable, including those on aircraft cabins [39], agricultural machines [40], military vehicles [41], pHRI activities [2], construction [42], and wheelchairs [43]. The comfort models are either deficient in human biomechanical knowledge or inappropriate for optimizing gestures. A comfort model based on energy expenditure will be developed in this study with the goal of improving the comfort of the human’s upper limbs in gesture interaction applications [44]. The model, that can determine the comfort of a human’s upper limbs in static or dynamic gestures, incorporates rich biomechanical information. The comfort model can be utilized to predict potential risks of discomfort in the muscles or injury, as well as gesture design optimization.

Additionally, excessive measurement inaccuracy will negatively impact the rate of gesture recognition, and it will affect both the usability and operational effectiveness of the gesture interaction. When measuring the human skeleton using depth stereoscopic vision, Zago [45] et al. did not take into account the measurement error resulting from the structural parameters and measurement positions of the stereoscopic depth model. Instead, they used a motion capture system to compensate for the human skeleton’s measurement error. In order to increase the measurement accuracy of the ToF depth camera, a noise filtering method [46] was applied to account for the impact of multipath error and ambient light error on the depth map. A theoretical error calculation equation based on an error propagation model [47] is proposed to rapidly establish accurate measurement systems that are capable of ensuring the accuracy of tube measurement systems based on multi-stereo vision. Through analysis of the depth measurement error’s effect factors, Wang [48] et al. found that the depth measurement error of binocular stereo vision is significantly influenced by the rotation angle errors and image feature extraction errors. However, this research only gives the guidelines, and does not give the specific parameter optimization method. Aiming at the problem of the loss of gesture features when the KLT tracker is occluded and large-scale rotation, Liu [49] uses a Kalman filter to predict the position of the gesture to improve the measurement precision of the gesture, but the study did not consider the depth information of gesture. Furthermore, some researchers look at the issue of the measurement accuracy from the perspectives of picture distortion correction [50] and technique comparison [51], but not from the structural features of the stereoscopic depth vision system. In order to reduce the binocular stereo vision measurement inaccuracy, the depth measurement error is derived by considering the structural parameters of the binocular stereo vision and the position of the measured object. We will aim to optimize the overall performance of the depth stereo measurement system.

The comfort and measurement accuracy concerns in gesture interaction applications were the main topics of this article. On the one hand, gestures’ comfort issues can result in a number of issues, including muscular fatigue, poor working efficiency, and a bad interaction experience. The usability of gesture interaction applications will be impacted by the measurement precision of motions, which will result in low gesture recognition rates. However, there is frequently a contradiction between measurement accuracy and comfort. Other targets’ performance can suffer if comfort is singly pursued. As a result, Our research will establish the comfort model and measurement precision model of gestures in light of the coordination problem of gestures in terms of the comfort and measurement precision, and will use multi-objective optimization methods to calculate the optimal design variables for improving the level of comfort, operating efficiency, experience, and usability of gesture interactive applications.

## 2. Gesture Comfort Modeling

An essential metric for assessing the ergonomics of the human upper limbs is comfort. It is very important to study the gesture design theory and techniques to lower operator risk and fatigue, and to enhance human–computer interaction. In order to optimize gestures, a model of gesture comfort based on muscle mechanical energy expenditure and efficiency was developed in the article.

### 2.1. Muscle Mechanical Energy Expenditure of Gesture

Human muscular energy expenditure is a crucial biomechanical parameter in the study of human biomechanics and has significant scientific implications in the areas of ergonomics, upper limb rehabilitation, muscle fatigue analysis, and human comfort assessment.

**Figure 1:**
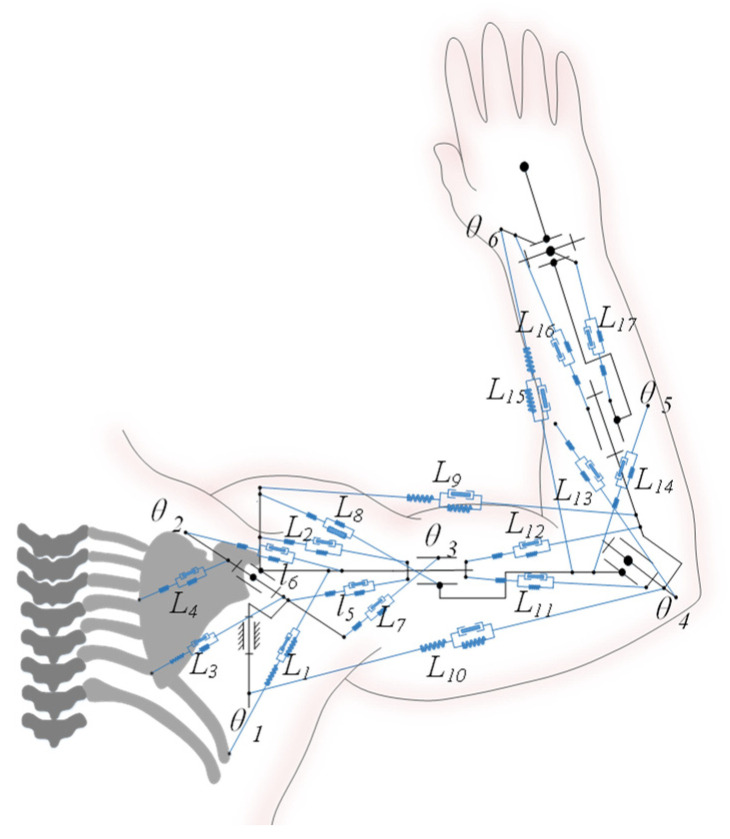
The schematic diagram of human upper limb musculoskeletal model. Note: 
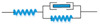
 The spring damping system represents the human muscle model.

In general, the human body uses two types of energy: muscle mechanical energy and calorie expenditure. Muscle contractions convert chemical energy into thermal and mechanical energy. However, because the primary role of muscles is to produce muscle power and the calorie expenditure is little, the amount of heat generated during muscle contraction is negligible. Figure 1 from the paper illustrates the human upper limb musculoskeletal model. The recommended comfort model based on the data from muscles and joints, CS can simulate the energy consumption of the gesture by taking into account six degrees of freedom and seventeen muscles.

However, for nonliving mechanical systems, there is no energy expenditure when the mechanical system is at rest. For the human body, whether the human upper limbs are performing a static or dynamic gesture, the muscles will produce mechanical energy expenditure. Therefore, according to the human upper limb musculoskeletal model as shown in Figure 1, the muscle mechanical energy expenditure model of the human upper limb is established. The muscle energy expenditure of the dynamic gesture is equal to the integral of the sum of the absolute value of the power of each joint in time, and the muscle energy expenditure of the static gesture is equal to the integral of the sum of the absolute value of muscle force in time. Then, the calculation formula of muscle energy expenditure of the gesture can be expressed as follows:

(1) MEEM=∫t1t2(∑i=16(Ti+θ˙i+Ti−θ˙i)+∑i=117Fimuscle_Li˙)dt,θ˙≠0∫t1t2∑i=117Fimuscle_dt,θ˙=0                                                      

where ${MEE}_{M}$ is the muscle energy consumption of gesture, $T_{i}^{+}$ and $T_{i}^{-}$ are the positive and negative joint torques caused by inertia, gravity, and ligament at the $i_{th}$ joint. ${\dot{\theta }}_{i}$ is joint angle velocity at the $i_{th}$ joint. Fimuscle_ and $\dot{L_{i}}$ are the muscle force and muscle length of the human upper limb at the ith joint.

Generally, muscle efficiency is not constant, but changes with the state of muscle contraction. Its value depends on the load and contraction velocity of the muscle. An appropriate muscle load and contraction velocity can maximize mechanical efficiency, but the muscle load and contraction rate are not fixed; they depend on the nerve stimulation state of the muscle. Therefore, the mechanical efficiency of muscles is also an important indicator to evaluate the ergonomics of human muscles. Then, the muscle mechanical efficiency of the gesture can be calculated from the ratio of muscle mechanical work to muscle mechanical energy consumption. The calculation formula is as follows:

(2) $$ME=\frac{W_{M}}{{MEE}_{M}}$$

(3) $$W_{M}=\int_{t_{1}}^{t_{2}}\sum_{i=1}^{6}\tau_{i}{\dot{\theta }}_{i}dt$$

where ME is the muscle mechanical efficiency of the human upper limbs, $W_{M}$ is the muscle mechanical work of the human upper limbs, $\tau_{i}$ is the joint net torque of the human upper limb at the $i_{th}$ joint.

### 2.2. Comfort Model of Gesture

The muscle mechanical energy expenditure of the gesture can reflect the load level of the active and passive muscles of the gesture, and the muscle mechanical efficiency can reflect the efficiency level of the muscles of the gesture. This paper establishes a gesture comfort model based on the muscle mechanical energy expenditure and mechanical efficiency through linear weighted combination. According to the habit of comfort evaluation in ergonomics, the comfort score of the gesture is set between 0 and 10. The smaller the score, the better the comfort. The calculation formula of the gesture comfort model is as follows:

(4) $$CS=k(w_{1}\frac{MEE}{{MEE}_{max}}+w_{2}\frac{W_{M}}{MEE})$$

where the CS is the comfort model of the gesture; k is the constant and k = 10; $w_{1}$ and $w_{2}$ are the weight coefficients of the MEE and ME; ${MEE}_{max}$ is the maximum energy consumption when the human upper limbs feel fatigue.

In summary, the comfort model of the gesture can analyze the comfort of static or dynamic gestures. In addition to MEE, the model also considers the impact of ME on comfort. MEE contains rich biomechanical information of the upper limbs’ movement posture, muscle strength, inertial force, ligament restraint force, muscle mechanical energy consumption and efficiency. It reflects the muscle energy consumption of the human upper limbs during movement. The greater the energy consumption, the more fatigue. The smaller the energy consumption, the more comfort. ME is the ratio of work $W_{M}$ to MEE, which reflects the muscles’ efficiency of the human upper limb. The higher the efficiency, the higher the utilization rate of the muscle, and the lower the efficiency, the lower the utilization rate of the muscle.

## 3. Measurement Precision Modeling

### 3.1. Depth Stereo Measurement Model

**Figure 2:**
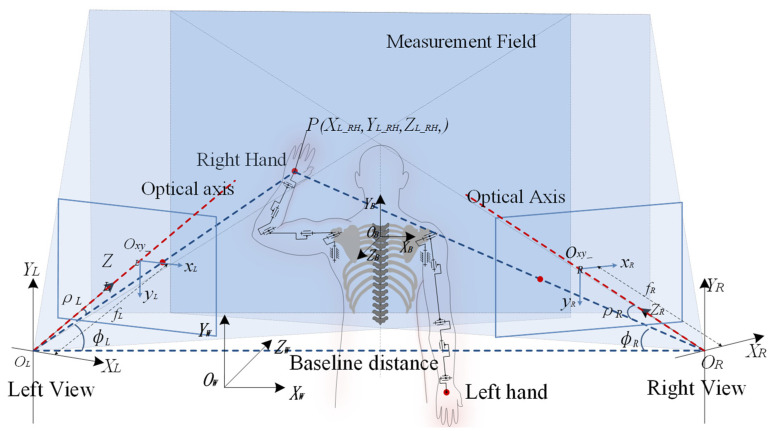
Depth stereo measurement model of gesture features (note:$O_{L}\left(X_{L},Y_{L},Z_{L}\right)$ and $O_{R}\left(X_{R},Y_{R},Z_{R}\right)$ are the left and right view coordinate systems of the depth stereo measurement model, $O_{B}\left(X_{B},Y_{B},Z_{B}\right)$ is human body coordinate system, $O_{xy\_L}\left(x_{L},y_{L}\right)$ and $O_{xy\_R}\left(x_{R},y_{R}\right)$ are the projection plane coordinate systems for the left and right view; the blue overlapping area is the measurement field of view; $f_{L}$ and $f_{R}$ are the focal lengths of the left and right view; $\phi_{l}$ and $\phi_{R}$ are the angles between the central axis and baseline; $\rho_{l}$ and $\rho_{R}$ are the projection angles of the left and right view; B is the baseline distance).

Depth stereo measurement is based on the principle of parallax, and obtains the depth value by comparing the same feature points in two projection planes. The depth stereo measurement precision will directly affect the recognition precision and efficiency of the gesture, and affect the level of ergonomics and interactive experience in gesture interaction applications. According to the principle of depth stereo measurement, the depth stereo measurement model is composed of the relative position relationship of the left/right view of the depth stereo measurement system, the projection angle, the angle relative to the optical axis, the focal length, and the position of the measurement object. The depth stereo measurement model of gesture features is shown in Figure 2.

In order to build the depth stereo measurement model, one first needs to establish a perspective relationship between the projection plane coordinate and the view coordinate of the gesture features. The formula is as follows:

(5) $$z_{L\_H}\left[\begin{array}{c} x_{L\_RH}\\ y_{L\_RH}\\ 1 \end{array}\right]=\left[\begin{array}{ccc} f_{L} & 0 & 0\\ 0 & f_{L} & 0\\ 0 & 0 & 1 \end{array}\right]\left[\begin{array}{c} X_{L\_RH}\\ Y_{L\_RH}\\ Z_{L\_H} \end{array}\right]$$

(6) $$z_{R\_H}\left[\begin{array}{c} x_{R\_RH}\\ y_{R\_RH}\\ 1 \end{array}\right]=\left[\begin{array}{ccc} f_{R} & 0 & 0\\ 0 & f_{R} & 0\\ 0 & 0 & 1 \end{array}\right]\left[\begin{array}{c} X_{R\_RH}\\ Y_{R\_RH}\\ Z_{R\_RH} \end{array}\right]$$

where the $\left(x_{L\_RH},y_{L\_RH},z_{L\_RH}\right)$ are the coordinates of the gesture features in coordinate system $O_{xy\_L}$; $\left(x_{R\_RH},y_{R\_RH},z_{R\_RH}\right)$ are the coordinates of the gesture features in coordinate system $O_{xy\_R}$; $\left(X_{L\_RH},Y_{L\_RH},Z_{L\_RH}\right)$ are the coordinates of the gesture features in coordinate system $O_{L}$; $\left(X_{R\_RH},Y_{R\_RH},Z_{R\_RH}\right)$ are the coordinates of the gesture features in coordinate system $O_{R}$; $f_{L}$ and $f_{R}$ are the focal length.

The relationship between $\left(X_{L\_RH},Y_{L\_RH},Z_{L\_RH}\right)$ and $\left(X_{R\_RH},Y_{R\_RH},Z_{R\_RH}\right)$ can be expressed by the spatial homogeneous transformation matrix $M_{LR}$:

(7) $$\left[\begin{array}{c} X_{R\_RH}\\ Y_{R\_RH}\\ Z_{R\_RH} \end{array}\right]=\left[\begin{array}{cccc} r_{1} & r_{2} & r_{3} & t_{x}\\ r_{4} & r_{5} & r_{6} & t_{y}\\ r_{7} & r_{8} & r_{9} & t_{z} \end{array}\right]\left[\begin{array}{c} X_{L\_RH}\\ Y_{L\_RH}\\ Z_{L\_RH}\\ 1 \end{array}\right]=M_{LR}\left[\begin{array}{c} X_{L\_RH}\\ Y_{L\_RH}\\ Z_{L\_RH}\\ 1 \end{array}\right]$$

where $M_{LR}$ is the spatial homogeneous transformation matrix between the coordinate system $O_{L}$ and $O_{R};r_{i}(i=1\sim 9)$ are the elements of the rotation matrix; $t_{x},\;t_{y},t_{z}$ are the elements of the translation vector.

Then, the relationship between the coordinate system $O_{xy\_L}$ and $O_{xy\_R}$ can be expressed as follows:

(8) $$\frac{Z_{R\_RH}}{z_{L\_RH}}\left[\begin{array}{c} x_{R\_RH}\\ y_{R\_RH}\\ 1 \end{array}\right]=\left[\begin{array}{cccc} f_{R}r_{1} & f_{R}r_{2} & f_{R}r_{3} & f_{R}t_{x}\\ f_{R}r_{4} & f_{R}r_{5} & f_{R}r_{6} & f_{R}t_{y}\\ r_{7} & r_{8} & r_{9} & t_{z} \end{array}\right]\left[\begin{array}{c} Z_{L\_RH}x_{L\_RH}/f_{L}\\ Z_{L\_RH}y_{L\_RH}/f_{L}\\ Z_{L\_RH}\\ 1 \end{array}\right]$$

Therefore, the three-dimensional coordinates of the gesture features in the right view coordinate system can be expressed as follows:

(9) $$\left\{\begin{array}{c} X_{L\_RH}=Z_{L\_RH}x_{L\_RH}/f_{L}\\ Y_{L\_RH}=Z_{L\_RH}y_{L\_RH}/f_{L}\\ Z_{L\_RH}=\frac{f_{L}(f_{R}t_{x}-x_{R_{1}}t_{z})}{x_{R\_RH}(r_{7}x_{L\_RH}+r_{8}y_{L\_RH}+f_{L}r_{9})-f_{R}(r_{1}x_{L\_RH}+r_{2}y_{L\_RH}+f_{L}r_{3})} \end{array}\right.$$

### 3.2. Measurement Precision Model

The efficiency of the gesture interaction is impacted by the depth stereo measurement precision, which also influences the measurement precision of the gesture features. In order to improve the measurement accuracy of the gesture features, the paper established a depth stereo measurement error model. In order to simplify the complexity of the depth stereo measurement model, it is assumed that the left and right view are placed horizontally and at the same height, and the coordinate origin of the depth stereo measurement model is the center position of the left view. Then, the simplified three-dimensional coordinates of the gesture features in the right view coordinate system can be expressed as follows:

(10) $$\left\{\begin{array}{c} X_{L\_RH}=\frac{BCOS(\rho_{l}+\phi_{l})}{COS\left(\rho_{L}+\phi_{L}\right)+cos(\rho_{R}+\phi_{R})}\;\;\;\;\;\;\;\;\;\;\;\;\;\;\;\;\;\;\;\;\;\;\;\;\;\;\;\;\;\;\;\;\;\\ Y_{L\_RH}=y_{L\_RH}\frac{Z_{H}sin(\rho_{l})}{f_{L}sin(\rho_{L}+\phi_{L})}=y_{R\_RH}\frac{Z_{H}sin(\phi_{R})}{f_{R}sin(\rho_{R}+\phi_{R})}\\ Z_{L\_RH}=\frac{B}{COS\left(\rho_{L}+\phi_{L}\right)+cos(\rho_{R}+\phi_{R})}\;\;\;\;\;\;\;\;\;\;\;\;\;\;\;\;\;\;\;\;\;\;\;\;\;\;\;\;\;\;\;\;\; \end{array}\right.$$

In order to analyze the influence of the parameters of the depth stereo measurement model on the precision, the partial derivative of Equation (10) can be obtained:

(11) $$\left\{\begin{array}{c} \frac{\partial X_{L\_RH}}{\partial x_{L\_RH}}=-\frac{{Z_{L\_RH}}^{2}}{Bf_{L}}\frac{\cos\left(\rho_{R}+\phi_{R}\right)}{{Sin}^{2}\left(\rho_{L}+\phi_{L}\right)}\cos^{2}\left(\rho_{L}\right)\\ \frac{\partial X_{L\_RH}}{\partial x_{R\_RH}}=-\frac{{Z_{L\_RH}}^{2}}{Bf_{R}}\frac{\cos\left(\rho_{L}+\phi_{L}\right)}{{Sin}^{2}\left(\rho_{R}+\phi_{R}\right)}\cos^{2}\left(\rho_{R}\right) \end{array}\right.$$

(12) $$\left\{\begin{array}{c} \frac{\partial Z_{L\_RH}}{\partial x_{L\_RH}}=-\frac{{Z_{L\_RH}}^{2}}{Bf_{L}}\frac{\cos^{2}\left(\rho_{L}\right)}{{Sin}^{2}\left(\rho_{L}+\phi_{L}\right)}\\ \frac{\partial Z_{L\_RH}}{\partial x_{R\_RH}}=-\frac{{Z_{L\_RH}}^{2}}{Bf_{R}}\frac{\cos^{2}\left(\rho_{R}\right)}{{Sin}^{2}\left(\rho_{R}+\phi_{R}\right)} \end{array}\right.$$

(13) $$\left\{\begin{array}{c} \frac{\partial Y_{L\_RH}}{\partial x_{L\_RH}}=\frac{Y_{L\_RH}Z_{L\_RH}}{Bf_{L}}\frac{\cos^{2}\left(\rho_{L}\right)}{{Sin}^{2}\left(\rho_{L}+\phi_{L}\right)}\\ \frac{\partial Y_{L\_RH}}{\partial x_{R\_RH}}=\frac{Y_{L\_RH}Z_{L\_RH}}{Bf_{R}}\frac{\cos^{2}\left(\rho_{R}\right)}{{Sin}^{2}\left(\rho_{R}+\phi_{R}\right)} \end{array}\right.$$

(14) $$\left\{\begin{array}{c} \frac{\partial Y_{L\_RH}}{\partial y_{L\_RH}}=\frac{Z_{L\_RH}}{f_{L}}\frac{Sin\left(\rho_{L}\right)}{Sin\left(\rho_{L}+\phi_{L}\right)}\\ \frac{\partial Y_{L\_RH}}{\partial y_{R\_RH}}=\frac{Z_{L\_RH}}{f_{R}}\frac{Sin\left(\rho_{R}\right)}{Sin\left(\rho_{R}+\phi_{R}\right)} \end{array}\right.$$

Generally, the average projection error ${∆}_{xy}$ of the depth stereo measurement model can be obtained by calibration, then the average error of the depth stereo measurement model in the left/right view coordinate system can be calculated as follows:

(15) $$\left\{\begin{array}{c} ∆X_{L}={∆}_{xy}dx,\;\;\;\;∆Y_{L}={∆}_{xy}dx\\ ∆X_{R}={∆}_{xy}dy,\;\;\;\;∆Y_{R}={∆}_{xy}dy \end{array}\right.$$

According to Figure 2, the projection angles on the left/right view are $\rho_{l}$ and $\rho_{R}$ and can be calculated as follows:

(16) $$\left\{\begin{array}{c} \rho_{l}=atan(Z_{L\_H},\sqrt{{X_{L\_H}}^{2}+{Y_{L\_H}}^{2}})\\ \rho_{R}=\rho_{l} \end{array}\right.$$

Then, the depth stereo measurement error in the X/Y/Z direction can be expressed as follows:

(17) $$\left\{\begin{array}{c} {∆X}_{RH}=\sqrt{{\left(\frac{\partial X_{L\_RH}}{\partial x_{LR\_H}}∆X_{L}\right)}^{2}+{\left(\frac{\partial X_{L\_RH}}{\partial x_{R\_RH}}∆X_{R}\right)}^{2}}\\ ∆Y_{RH}=\sqrt{{\left(\frac{\partial Y_{L\_RH}}{\partial x_{L\_RH}}∆X_{L}\right)}^{2}+{\left(\frac{\partial Y_{L\_RH}}{\partial x_{R\_RH}}∆X_{R}\right)}^{2}+{\left(\frac{\partial Y_{L\_RH}}{\partial y_{L\_RH}}∆Y_{L}\right)}^{2}+{\left(\frac{\partial Y_{L\_RH}}{\partial y_{R\_RH}}∆Y_{R}\right)}^{2}}\\ {∆Z}_{RH}=\sqrt{{\left(\frac{\partial Z_{L\_RH}}{\partial x_{L\_RH}}∆X_{L}\right)}^{2}+{\left(\frac{\partial Z_{L\_RH}}{\partial x_{R\_RH}}∆X_{R}\right)}^{2}} \end{array}\right.$$

Therefore, the depth stereo measurement precision model can be expressed as follows:

(18) $$\begin{array}{cc} {∆P}_{H} & =\sqrt{∆X_{H}^{2}+∆Y_{H}^{2}+∆Z_{H}^{2}}\\ & =\sqrt{\begin{array}{c} {\left(\frac{\partial X_{L\_RH}}{\partial x_{L_{RH}}}∆X_{L}\right)}^{2}+{\left(\frac{\partial X_{L\_RH}}{\partial x_{R_{RH}}}∆X_{R}\right)}^{2}+{\left(\frac{\partial Y_{L\_RH}}{\partial x_{L\_RH}}∆X_{L}\right)}^{2}+{\left(\frac{\partial Y_{L\_RH}}{\partial x_{R\_RH}}∆X_{R}\right)}^{2}+\\ {\left(\frac{\partial Y_{L\_RH}}{\partial y_{L\_RH}}∆Y_{L}\right)}^{2}+{\left(\frac{\partial Y_{L\_RH}}{\partial y_{R\_RH}}∆Y_{R}\right)}^{2}+{{∆Z}_{H}}^{2}{\left(\frac{\partial Z_{L\_RH}}{\partial x_{L\_RH}}∆X_{L}\right)}^{2}+{\left(\frac{\partial Z_{L\_RH}}{\partial x_{R\_RH}}∆X_{R}\right)}^{2} \end{array}} \end{array}$$

where $\left({∆X}_{H},{∆Y}_{H},{∆Z}_{H}\right)$ are the depth stereo measurement error in the X/Y/Z direction; $∆X_{L},∆Y_{L}$,$∆X_{R},∆Y_{R}$ are the average projection errors in the projection plane of the left/right view; ${∆P}_{H}$ is the depth stereo measurement precision model.

The binocular stereo vision parameter may be guided by the precision analysis, in accordance with the depth stereo measurement precision model. This can guide us how to choose the lens ($f_{L}$ and $f_{R}$) of the camera, so as to obtain a higher measurement accuracy. In addition, the appropriate baseline distance B and the distance between the camera and the measured object can be given. Even the position with the lowest measurement accuracy can be given, so that this position can be avoided as much as possible in practical operation.

## 4. Multi-Objective Optimization Method for Gestures

### 4.1. Multi-Objective Optimization Model

The usability of gesture interaction applications is influenced by a number of significant elements, including the user comfort and measurement accuracy. The ease of use and measurement accuracy of the gestures will influence how easily the human upper limb muscles fatigue and how well they operate. However, there is frequently a conflict between the two performances of the comfort and measurement accuracy. If there is, it can result in additional performance deterioration. In order to maximize co-optimization, it is required to coordinate and compromise between the performances of the comfort and measurement accuracy. In order to reduce muscle fatigue, increase operation efficiency, and enhance the interactive experience of the gesture in interactive applications, this paper proposed a multi-objective optimization method of the gesture based on comfort and measurement precision. It uses this method to calculate the optimal design variables that can make the gesture achieve the co-optimization.

**Figure 3:**
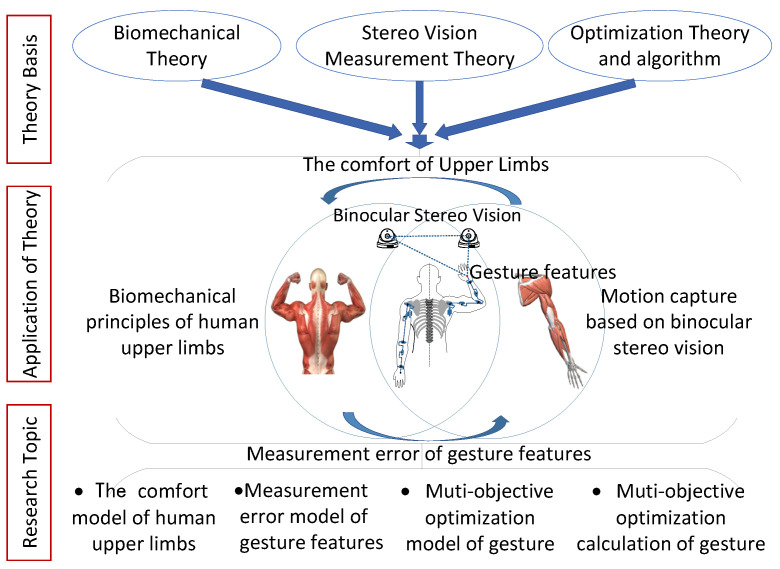
Schematic diagram of multi-objective optimization method of gestures.

Schematic diagram of the multi-objective optimization method of the gesture, as shown in Figure 3. The biomechanics theory of human upper limbs, the depth stereo measurement theory, the multi-objective optimization theory and algorithm, as well as other related theories and research, were all taken into consideration when developing the multi-objective optimization method of gestures suggested in this work. Research on multi-objective gesture optimization is useful for improving the ergonomics of gesture interaction applications, including comfort, operational effectiveness, and interactive experience.

According to the proposed gesture comfort model CS and measurement precision model ${∆P}_{H}$, the two single objective functions of the multi-objective optimization based on the gesture comfort and measurement error are expressed as follows:

(19) $$MinJ_{CS}=k(w_{MEE}\frac{MEE}{{MEE}_{max}}+w_{ME}\frac{W_{M}}{MEE})$$

(20) $$MinJ_{P_{H}}=\sum {∆P}_{H}=\sum \sqrt{∆X_{H}^{2}+∆Y_{H}^{2}+∆Z_{H}^{2}}$$

In order to simplify the multi-objective optimization objective function of the gesture and reduce the calculation amount, the two objective functions are transformed into a single objective function through the linear weighting method:

(21) $$MinJ=fmincon(w_{1}{\;J}_{CS}+w_{2}{\;J}_{P_{H}})$$

(22) $$s.t\left\{\begin{array}{c} {x_{B\_RH\_Min}\leq x}_{B\_RH}\leq x_{B\_RH\_Max}\\ {y_{B\_RH\_Min}\leq y}_{B\_RH}\leq y_{B\_RH\_Max}\\ {z_{B\_RH\_Min}\leq z}_{B\_RH}\leq z_{B\_RH\_Max} \end{array}\right.$$

where J is the multi-objective optimization objective function based on the comfort of gestures and measurement precision; $w_{1}$ and $w_{2}$ are the weight coefficient of the ${\;J}_{CS}$ and ${\;J}_{P_{H}}$; fmincon is the calculated functions for multi-objective optimization; $\left(x_{B\_RH},y_{B\_RH},z_{B\_RH}\right)$ are the coordinates of the gesture features as optimization variables.

### 4.2. Multi-Objective Optimization Calculation

**Figure 4:**
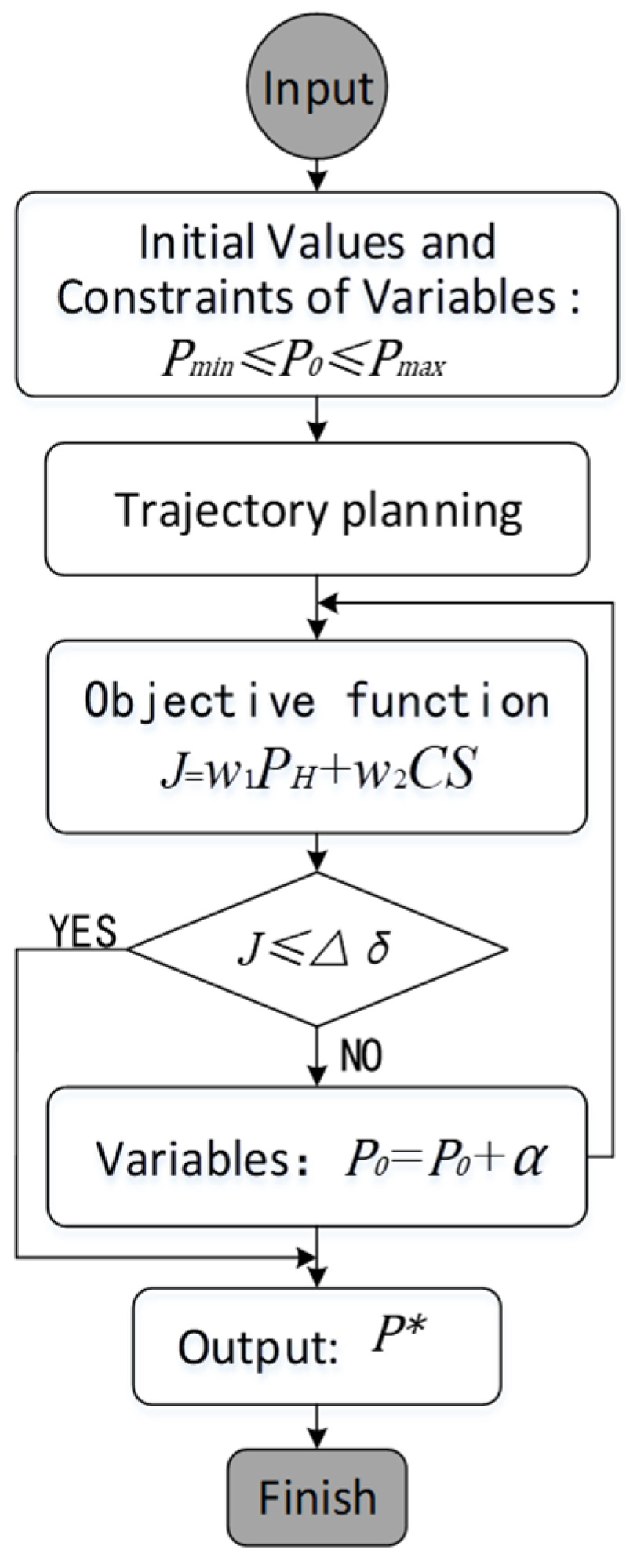
The flow chart of multi-objective optimization calculation for gesture.

The coordinates of the gesture features $\left(x_{B\_RH},y_{B\_RH},z_{B\_RH}\right)$ were used as optimization variables and gesture-based circular trajectories were optimized in accordance with the established multi-objective optimization model of the gesture based on the comfort and measurement error, so that the gesture achieved an optimal performance in both comfortable and measurement error. Figure 4 depicts the flow chart of a multi-objective optimization computation for a gesture based on the measurement accuracy and comfort.

The nonlinear programming solver is used to perform multi-objective optimization calculations for the gesture based on the comfort and measurement errors. Firstly, give the initial values $P_{0}$ and variable constraints $P_{min}\leq P_{0}\leq P_{max}$; secondly, use the trajectory planning algorithm to calculate the human upper limb movement trajectory; thirdly, calculate the objective function J of the gesture multi-objective optimization model; fourthly, determine whether the objective function meets the iteration stop condition J≤∆δ, if it is satisfied, output the current optimal solution $P^{*}$, if not, continue to the next step; fifthly, modify the initial value of the variable $P_{0}=P_{0}+\alpha$ with a given step size α along the search direction, and return to the third step for iterative calculation; finally, the judgment condition is met, and the iteration is terminated.

### 4.3. Case Analysis and Results of Multi-Objective Optimization of Gestures

In order to verify the effectiveness of the proposed method, the gesture of the circular trajectory is exemplified in the work undertaken by the multi-objective optimization. According to the position of the circle center $P_{B\_RHO}\left(x_{B\_RHO},y_{B\_RHO},z_{B\_RHO}\right)$ and radius $r_{RH}$, a continuous circular trajectory can be calculated using the trajectory planning algorithm, so that the gesture comfort and measurement precision can be calculated. Therefore, the multi-objective optimization model of the gesture based on the circular trajectory can be expressed as follows:

(23) $$MaxJ=fmincon(w_{1}{\;J}_{CS}+w_{2}{\;J}_{P_{H}})$$

(24) $$s.t\left\{\begin{array}{c} \begin{array}{c} -0.6\leq x_{B\_RHO}\leq 0.6\\ -0.6\leq y_{B\_RHO}\leq 0.6\\ 0\leq z_{B_{RHO}}\leq 0.6\\ 0.1\leq r_{RH}\leq 0.6 \end{array} \end{array}\right.$$

According to the movement habits of most people, this paper makes the following assumptions: gestures of circular trajectory make clockwise movements, and the initial position $P_{B\_RH1}\left(x_{B\_RH1},y_{B\_RH1},z_{B\_RH1}\right)$ and target position $P_{B\_RH2}\left(x_{B\_RH2},y_{B\_RH2},z_{B\_RH2}\right)$ of the circular trajectory are coincided and above the center of the circle. Then, the position of the center of the circle is equal to $P_{B\_RHO}\left(x_{B\_RHO},y_{B\_RHO},z_{B\_RHO}\right)$ $=\left(x_{B\_RH1},y_{B\_RH1}-r_{RH},z_{B\_RH1}\right)$. Therefore, the non-linear optimization function fmincon was used to solve the optimal solution until the convergence condition is met and the iteration is stopped.

**Figure 5:**
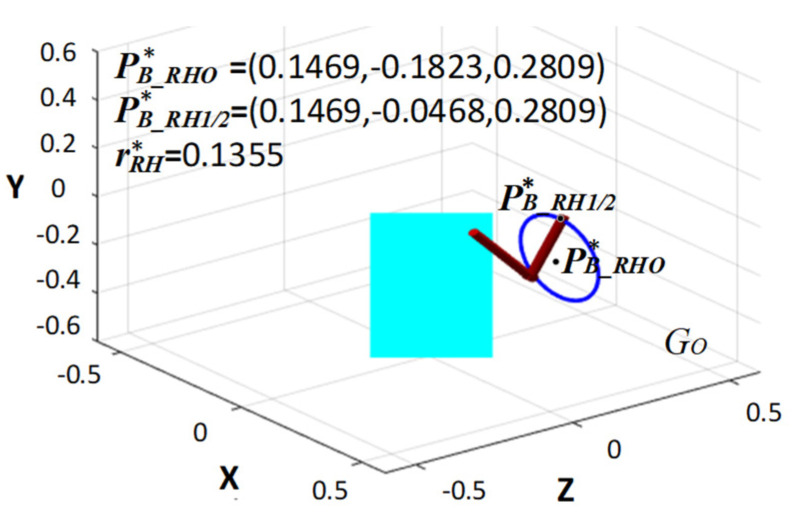
The multi-objective optimization results of gestures based on circular trajectories.

As shown in Table 1, the initial values of the position of the center of the circle were equal to $P_{B\_RHO}=(0,\;0,\;0.2)$ and the $r_{RH}$ is equal to 0.1; the optimal solutions of the position of the center of the circle were equal to $P_{B\_RHO}^{*}=(0.1469,-0.1823,\;0.2809)$ and the radius is equal to $r_{RH}^{*}=0.1355$. So, the optimal initial and target position of the circular trajectory are equal to $P_{B\_RH1/2}^{*}\left(x_{B\_RH1/2},y_{B\_RH1/2},z_{B\_RH1/2}\right)=(0.1469,-0.0468,\;0.2809)$. The result of the gesture optimization based on the circular trajectory is shown in Figure 5.

## 5. Discussion

To illustrate the effectiveness of the multi-objective optimization method for the gesture based on the comfort and measurement precision, the comparison and analysis are performed among the objective functions of ${\;J}_{CS},{\;J}_{P_{H}}$, and $J_{CP}$ based on the case analysis of the multi-objective optimization of gestures. The center coordinates ($x_{B\_RHo},y_{B\_RHo},z_{B\_RHo}$) and radius $r_{RH}$ are changed as the variables to analyze the change law of the objective functions.

**Figure 6:**
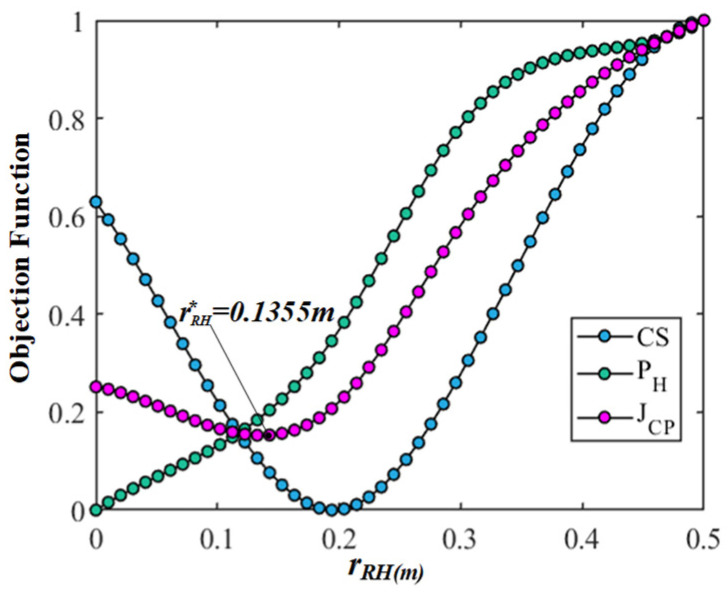
The results comparison and analysis of the objective functions corresponding to the radius $r_{RH}$ of the circular trajectory.

**Figure 7:**
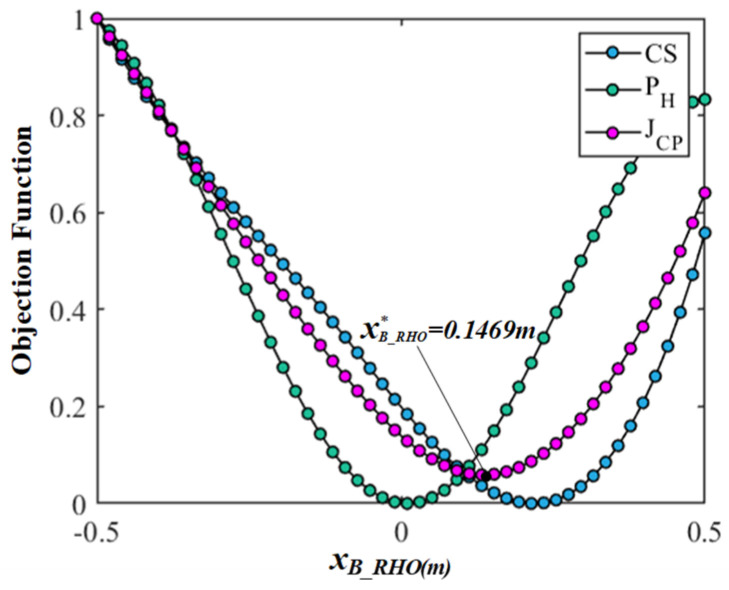
The results comparison and analysis of the objective functions corresponding to variable $x_{B\_RHO}$.

**Figure 8:**
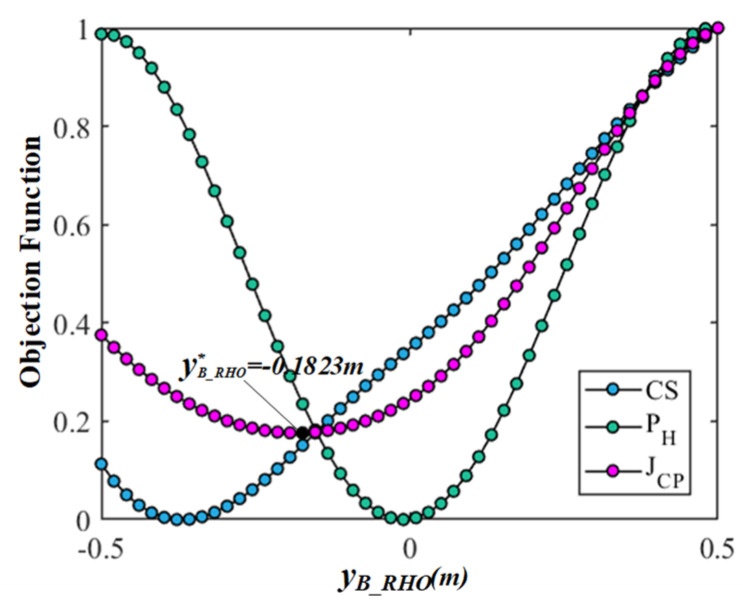
The results comparison and analysis of the objective functions corresponding to variable $y_{B\_RHO}$.

**Figure 9:**
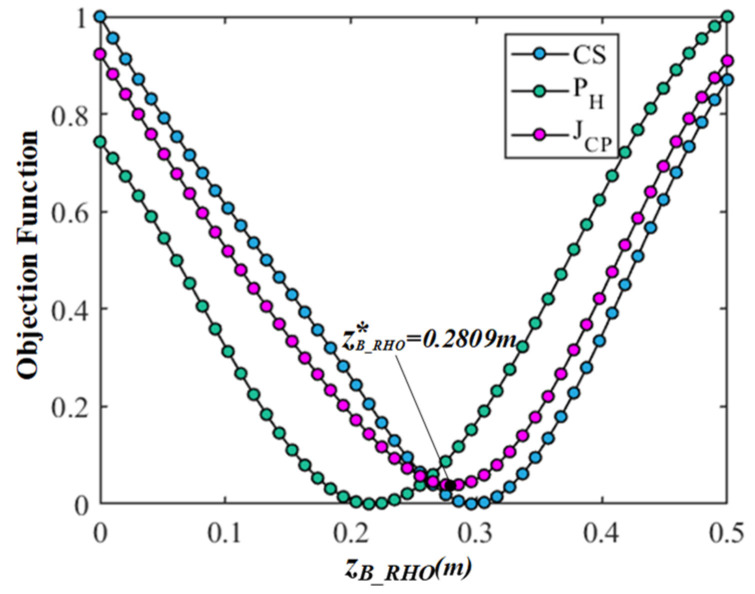
The results of comparison and analysis of objective functions corresponding to variable $z_{B\_RHO}$.

Firstly, in order to eliminate the difference and dimension among the ${\;J}_{CS},{\;J}_{P_{H}}$, and $J_{CP}$, the objective functions are transformed into dimensionless values between 0 and 1 through standardization. Then, all data indicators are in the same order of magnitude to solve the comparability among the objective functions, which is convenient for the weighting processing and comparative analysis and intuitively understanding the change law of each objective function corresponding to each variable. Finally, the results of the comparison and analyses based on the multi-objective optimization of the gesture are shown in Figure 6, Figure 7, Figure 8 and Figure 9. In the figures, the green, blue, and magenta curves represent the change law of the objective function of ${\;J}_{CS},{\;J}_{P_{H}}$, and $J_{CP}$ corresponding to different characteristic variables. Through the comparison and analysis, the effectiveness of the proposed method for optimizing the comprehensive performance of the gesture’s comfort and measurement precision is illustrated.

In Figure 6, according to the case analysis in the work undertaken by the multi-objective optimization based on gestures of circular trajectories, the center coordinates are set to $P_{B\_RHO}=(0.1469,-0.1823,\;0.2809)$, and the radius $r_{RH}$ of the circular trajectory varies from 0 to 0.5 m. The figure shows that the curve of the objective function ${\;J}_{CS}$ increases as the radius $r_{RH}$ increases. The curve of the objective function ${\;J}_{P_{H}}$ is inversely parabolic as the radius $r_{RH}$ increases. The trend of the objective function ${\;J}_{CS}$ and ${\;J}_{P_{H}}$ are partially conflicting and partially identical. The objective function $J_{CP}$ is weighted by ${\;J}_{CS}$ and ${\;J}_{P_{H}}$, and the optimal radius of the circular trajectory can be obtained through the multi-objective optimization calculation $r_{B\_RHO}^{*}=0.1355\;m$. The comparison result illustrates that for the gesture of circular trajectories, the radius $r_{RH}$ will affect the comfort and measurement precision of the gesture, especially when the radius is too large. The objective function $J_{CP}$ can combine the comprehensive performance of the gesture comfort and measurement precision.

In Figure 7, the center coordinates of the circular trajectory are set to $P_{B\_RHO}=(x_{B\_RHO},-0.1823,\;0.2809)$, the coordinate $x_{B\_RHO}$ determines the position of the gesture on the left and right sides of the body and varies from −0.5 to 0.5 m. The radius $r_{B\_RHO}$ is equal to 0.1355 m. Figure 8 shows the experimental data on the objective function of ${\;J}_{CS},{\;J}_{P_{H}}$, and $J_{CP}$ corresponding to the variable $x_{B\_RHO}$, respectively. The objective function ${\;J}_{CS}$ and ${\;J}_{P_{H}}$ show the same trend, but there are still obvious differences. The $x_{B\_RHO}$ position corresponding to the optimal measurement precision is in the middle of the body, and the optimal comfort is at the position of $x_{B\_RHO}=0.2143\;m$. The objective function $J_{CP}$ compromises the performance of ${\;J}_{CS}$ and ${\;J}_{P_{H}}$ and obtains the optimal center coordinate $x_{B\_RHO}^{*}=0.1469\;m$. The comparison result shows that there is no significant difference in the trend of the objective function ${\;J}_{CS}$ and ${\;J}_{P_{H}}$, but the center coordinate $x_{B\_RHO}$ has a significant impact on the comfort and measurement precision.

In Figure 8, the center coordinates of the circular trajectory are set to $P_{B\_RHO}=(0.1355,y_{B\_RHO},0.2809)$, and the coordinate $y_{B\_RHO}$ determines the height of the position of gesture and varies from −0.5 to 0.5 m. The radius $r_{B\_RHO}$ equal to 0.1355 m. The curve of objective function ${\;J}_{CS}$ increase with the change in $y_{B\_RHO}$, but the curve of objective function $P_{H}$ is parabolic with the change in $y_{B\_RHO}$. It can be observed that the differences should be considerable between ${\;J}_{CS}$ and ${\;J}_{P_{H}}$, especially when the $y_{B\_RHO}$ is in the range of −0.5 to 0 m. The objective function $J_{CP}$ reconciles the conflict between the ${\;J}_{CS}$ and ${\;J}_{P_{H}}$ and obtains the optimal center coordinate $y_{B\_RHO}^{*}=-0.1823\;m$. The comparison results show that the center coordinate $y_{B\_RHO}$ has a different impact on ${\;J}_{CS}$ and ${\;J}_{P_{H}}$, but the objective function $J_{CP}$ makes a compromise and achieve a common optimal between the comfort and measurement precision of gesture.

In Figure 9, the center coordinates of the circular trajectory are set to $P_{B\_RHO}=(0.1355,-1823,z_{B\_RHO})$, and the coordinate $z_{B\_RHO}$, that determines the distance of the gesture from the body, varies from 0 to 0.5 m. The radius $r_{B\_RHO}$ is equal to 0.1355 m. It can be observed that the objective functions ${\;J}_{CS}$ and ${\;J}_{P_{H}}$ show a high consistency, but there are still slight differences. Further improvement can still be made by the multi-objective optimization, and the optimal center coordinate $z_{B\_RHO}^{*}$ can be obtained as equal to 0.1403 m. The comparison result shows that the trend of ${\;J}_{CS}$ and ${\;J}_{P_{H}}$ affected by the center coordinate $z_{B\_RHO}$ is relatively consistent, but the impact on the comfort and measurement precision cannot be ignored.

In summary, these results show that the objective functions ${\;J}_{CS}$ and ${\;J}_{P_{H}}$ based on the circular trajectory gestures are related to the radius $r_{B\_RHO}$ and the center coordinates $P_{B\_RHO}\left(x_{B\_RHO},y_{B\_RHO},z_{B\_RHO}\right)$. Through the proposed multi-objective optimization method based on the gesture comfort and measurement precision models, the $r_{B\_RHO}$ and $P_{B\_RHO}\left(x_{B\_RHO},y_{B\_RHO},z_{B\_RHO}\right)$ are optimized to an appropriate position. Among them, compared with parameters $x_{B\_RHO}$ and $z_{B\_RHO}$, the difference between the objective function ${\;J}_{CS}$ and ${\;J}_{P_{H}}$ affected by the parameters $r_{B\_RHO}$ and $y_{B\_RHO}$ is more significant. The objective function $J_{CP}$ integrates the differences between the ${\;J}_{CS}$ and ${\;J}_{P_{H}}$ by weighting, and improves the comprehensive performance of gestures in terms of the comfort and measurement precision.

## 6. Conclusions

The current study aims to address the inconsistency between the measurement accuracy and gesture comfort in gesture interaction. This paper proposes a multi-objective optimization method based on the gesture comfort and measurement precision. Firstly, the gesture comfort CS is modeled by the muscle energy expenditure of the human upper limb. The CS provides an intuitive indicator ${\;J}_{CS}$ to predict which act has the higher risk of fatigue or injury for joints and muscles, so as to reduce operators’ fatigue and extend their working hours. Secondly, the depth stereo measurement precision ${∆P}_{H}$ was modeled by the measurement error. The ${∆P}_{H}$ provides an indicator ${\;J}_{P_{H}}$ to evaluate the operation efficiency of the gesture interaction. Then, we proposed a multi-objective optimization model $J_{CP}$ based on the ${\;J}_{CS}$ and ${\;J}_{P_{H}}$, that provides a method to achieve an optimal performance between the gesture comfort and measurement precision. Finally, a case analysis based on the circular trajectory gesture is implemented to verify the effectiveness of the multi-objective optimization method proposed in this paper. The comparison result shows both the consistency and the difference between the objective function ${\;J}_{CS}$ and ${\;J}_{P_{H}}$ corresponding to different parameters. The multi-objective optimization method of the gesture proposed in this paper effectively solves the inconsistency between the gesture comfort and measurement precision in gesture interaction. In general, the research in this paper is of great significance to the improvement of ergonomics and interaction efficiency in gesture interaction.

In the future, for robot teleoperation based on gesture interaction, the authors will carry out gesture design and use the multi-objective optimization method proposed in this paper to improve the ergonomics and operation efficiency of the gesture. Furthermore, the research will focus on the usability problem of gesture interaction, and comprehensively evaluate the satisfaction, comfort, effectiveness, operation efficiency, consistency, and interactive experience in gesture interaction through usability testing to verify the effectiveness of our work.

## Figures and Tables

**Table 1:** Initial values and optimal solutions of gesture optimization variable.

Variable Values	$\mathrm{Center}\;\mathrm{Position}\;\mathrm{of}\;\mathrm{Circle}\;P_{B\_RHO}$			Radius
	$x_{B\_RH0}$ (m)	$y_{B\_RH0}$ (m)	$z_{B\_RH0}$ (m)	$r_{RH}$ (m)
$\mathrm{Initial}\;\mathrm{values}\;P_{0}$	0	0	0.2	0.1
$\mathrm{Optimal}\;\mathrm{solutions}\;P^{*}$	0.1469	−0.1823	0.2809	0.1355

## Data Availability

All the data are available prior request to the authors.
